# An HPLC Method for Microanalysis and Pharmacokinetics of Marine Sulfated Polysaccharide PSS-Loaded Poly Lactic-*co*-Glycolic Acid (PLGA) Nanoparticles in Rat Plasma

**DOI:** 10.3390/md11041113

**Published:** 2013-04-02

**Authors:** Peng-Li Li, Chun-Xia Li, Yi-Ting Xue, Hai-Hua Li, Hong-Bing Liu, Xiao-Xi He, Guang-Li Yu, Hua-Shi Guan

**Affiliations:** Key Laboratory of Marine Drugs, Ministry of Education, Shandong Provincial Key Laboratory of Glycoscience and Glycotechnology, School of Medicine and Pharmacy, Ocean University of China, Qingdao 266003, China; E-Mails: pengli.li@163.com (P.-L.L.); xueyiting7365676@126.com (Y.-T.X.); shaixuan@ouc.edu.cn (H.-H.L.); liuhongb@ouc.edu.cn (H.-B.L.); hexiaoxi@ouc.edu.cn (X.-X.H.); glyu@ouc.edu.cn (G.-L.Y.); hsguan@ouc.edu.cn (H.-S.G.)

**Keywords:** microanalysis, pharmacokinetics, polysaccharide, nanoparticles, postcolumn fluorescence derivatization

## Abstract

This study was aimed at developing a sensitive and selective HPLC method with postcolumn fluorescence derivatization for the detection of propylene glycol alginate sodium sulfate (PSS) in rat plasma. Plasma samples were prepared by a simple and fast ultrafiltration method. PSS was extracted from rat plasma with d-glucuronic acid as internal standard. Isocratic chromatographic separation was performed on a TSKgel G2500 PWxL column with the mobile phase of 0.1 M sodium sulfate at a flow rate of 0.5 mL/min. Analyte detection was achieved by fluorescence detection (FLD) at 250 nm (excitation) and 435 nm (emission) using guanidine hydrochloride as postcolumn derivatizing reagent in an alkaline medium at 120 °C. The calibration curve was linear over a concentration range of 1–500 μg/mL, and the lower limit of detection (LLOD) was found to be 250 ng/mL. This validated method was applied successfully to the pharmacokinetic study of PSS and PSS-loaded poly lactic-*co*-glycolic acid (PLGA) nanoparticles (PSS-NP) in rat plasma after a single intravenous (PSS only) and oral administration (PSS and PSS-NP). Significant differences in the main pharmacokinetic parameters of PSS and PSS-NP were observed. The relative bioavailability of PSS-NP was 190.10% compared with PSS which shows that PSS-NP can improve oral bioavailability.

## 1. Introduction

**Figure 1:**
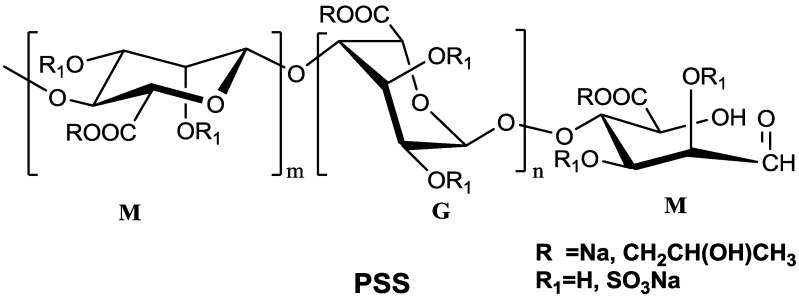
The general structure of propylene glycol alginate sodium sulfate (PSS).

Cardiovascular and cerebrovascular diseases, encompassing coronary artery disease, heart failure, acute myocardial infarction, arrhythmias *et al*., are the most common causes of morbidity and mortality in the world [1]. Propylene glycol alginate sodium sulfate (PSS) is a marine sulfated polysaccharide composed of β-d-mannuronic acid (M) and α-l-guluronic acid (G) (Figure 1). The average molecular weight of PSS is 11 KDa and the distribution width of the molecular weight is about 1.6. Systematic pharmacodynamics studies have shown that PSS has good anticoagulation, hypotensive activity, reducing blood viscosity and other functions [2].

PSS was first authorized for clinical applications in China in the 1980s. To date, the routes of administration of PSS have been oral tablets and clinical intravenous injection. However, there are some side effects of PSS with intravenous administration [3]. Furthermore, the oral bioavailability of PSS is low due to poor absorption. In recent years, a number of potential oral formulations of PSS have been developed to improve its oral bioavailability [4,5]. 

**Figure 2:**
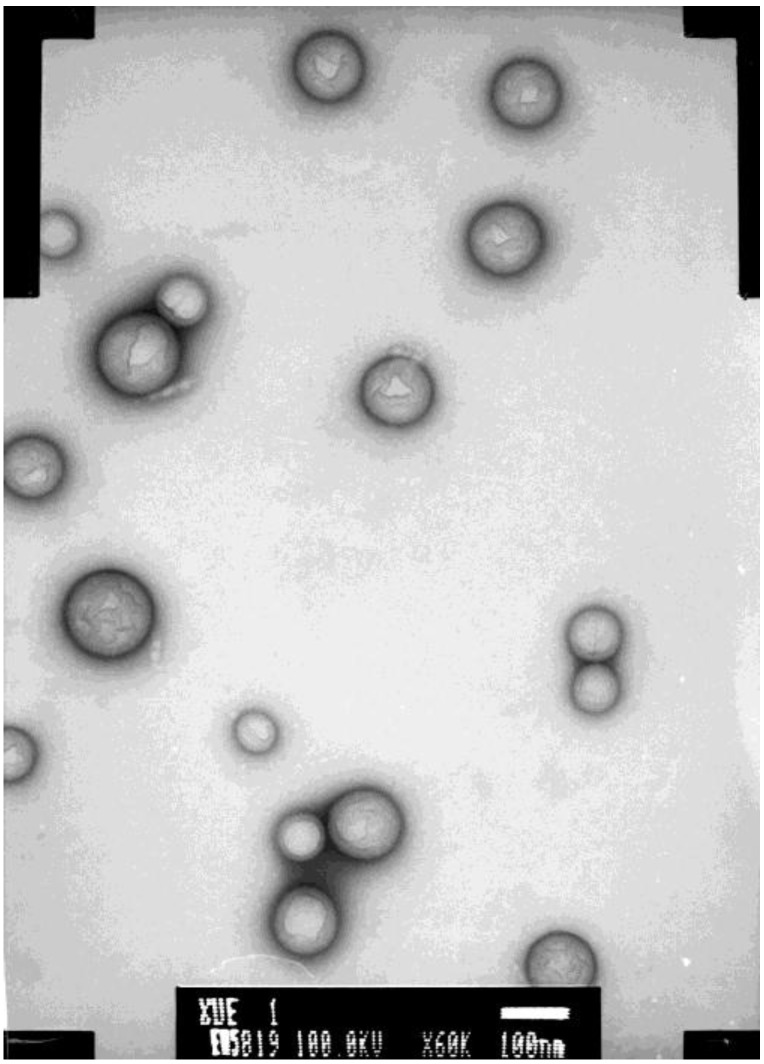
The morphology of PSS-loaded poly lactic-*co*-glycolic acid (PLGA) nanoparticles (PSS-NP) examined by transmission electron microscope (TEM).

Nanotechnology is one of the key technologies of the 21st century, in view of several notable relevant characteristics, such as the smaller size, higher capacity of loading hydrophilic drugs, and a better performance of controlled release, *etc.* [6,7,8]. Poly lactic-*co*-glycolic acid (PLGA), approved by the FDA, is extensively used as a pharmaceutical material because of its biocompatibility and biodegradability [9,10]. In order to improve the oral availability of PSS, we firstly prepared PSS-loaded PLGA nanoparticles (PSS-NP) by the double (W_1_/O/W_2_) emulsion and solvent evaporation methods and then their physicochemical characteristics, drug release *in vitro* were investigated [11]. Figure 2 shows the morphology of PSS-NP examined by transmission electron microscope (TEM). The average size of PSS-NP was 181.8 nm and the PDI was 0.012; the entrapment efficiency was 75.80% and the drug loading efficiency was 10.83%. In order to investigate the relative bioavailability of this new formulation, a method of pharmacokinetic study suitable for humans was explored in this study.

PSS possesses few chromophoric or fluorophoric groups. The sensitivities of the microanalysis of PSS in biological samples by measuring the refractive, scattering indexes or using colorimetric assays are relatively poor. Due to the limitations of traditional analysis techniques and methods, there are not many suitable methods to study the pharmacokinetic characteristics of PSS. The labeling method of tritium (^3^H) was firstly applied to early pharmacokinetic studies of PSS [12]. However, taking into account the radioactivity of ^3^H, it was unfit to use for the study of pharmacokinetic characters in people. Previously, we developed a fluorescent labeling method to microanalyze PSS and study its pharmacokinetic parameters with fluorescein isothiocyanate (FITC). The fluorescent labeling method was of high sensitivity and safety with relatively low cost. However, this method was complicated, time-consuming and also could not be applied to pharmacokinetic studies in people. 

In recent years, a high performance liquid chromatographic method with postcolumn fluorescence derivatization has been developed to determine sulfated carbohydrates in biological samples [13,14,15,16,17]. This method is convenient and automatic. Furthermore, it can be applied to study the pharmacokinetic characteristics of people.

In this study, a new high performance liquid chromatographic method with postcolumn fluorescence derivatization was developed to successfully analyze PSS in rat plasma after administration of PSS and PSS-loaded PLGA nanoparticles and to compare their pharmacokinetic behaviors. 

## 2. Results and Discussion

### 2.1. HPLC Method Development and Sample Handling

In the initial experiments, several attempts including the selections of columns, the fluorescence derivatization reagents and the internal standard (IS) were carried out to determine the analytes in plasma.

The column selection was based on the properties of PSS and relevant literature of similar polysaccharide drugs. Agilent Zorbax NH_2_ column (4.6 × 150 mm, Agilent, USA) was used firstly based on ion-exchange and molecular polarity of PSS but the peak shape and the separation efficiency were relatively poor. Based on size exclusion and distribution, TSKgel G3000PWxL and TSKgel G2500PWxL columns were tried, which are different in the measurement range of molecular weights. The glucose in plasma was separated excellently from PSS and the internal standard (IS) by the TSKgel G2500PWxL column, so it was chosen as the analytical column. Sodium sulfate (0.1 M) was chosen as the appropriate mobile phase after testing several mobile phases and the appropriate flow rate was established at 0.5 mL/min. Then benzamidine and guanidine hydrochloride were selected as fluorescence derivatization reagents as a check. The experiments showed that guanidine hydrochloride had high sensitivity compared with benzamidine. Therefore guanidine hydrochloride was used as the fluorescence derivatization reagent. d-glucuronic acid was chosen as the IS due to its similar chromatographic characteristics to PSS after also analyzing several oligosaccharides such as disaccharide.

In order to improve the sensitivity of the method, three derivatization reaction factors were tested: the concentration of guanidine hydrochloride, reaction temperature and the concentration of sodium hydroxide. As a result, 0.1 M guanidine hydrochloride, 120 °C and 0.5 M sodium hydroxide were chosen as the optimal reaction conditions. 

In addition, several methods were tried to pretreat the rat plasma. It was effective to remove blood protein and separate PSS from the endogenous substances in plasma by trichloroacetic acid precipitation and ultrafiltration centrifugation (30 kDa, MWCO). Compared with trichloroacetic acid precipitation, the peak signal strength of PSS was higher, and the baseline was relatively flat with ultrafiltration centrifugation. Therefore the method of ultrafiltration centrifugation was chosen to pretreat rat plasma. 

### 2.2. HPLC Method Validation

#### 2.2.1. Specificity

**Figure 3:**
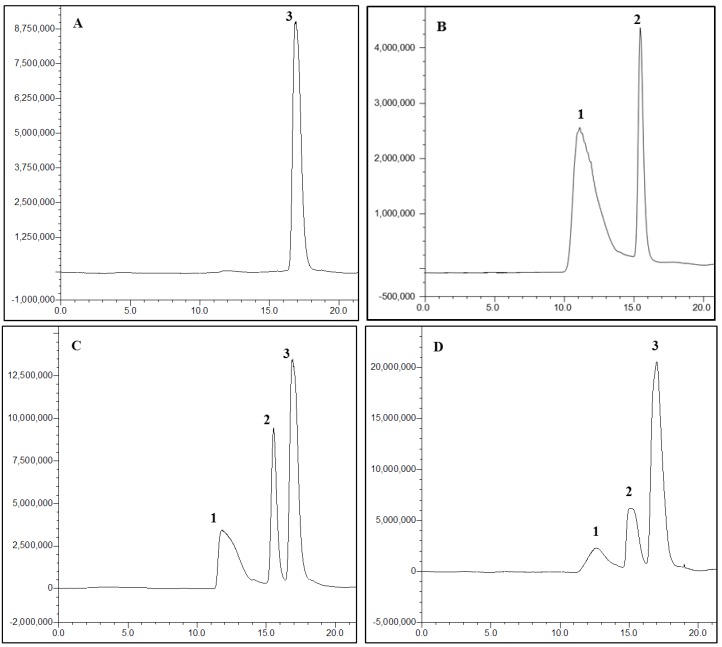
Chromatograms of (**A**) blank rat plasma, (**B**) 1 mg/mL PSS and 1 mg/mL d-glucuronic acid dissolved in the mobile phase, (**C**) blank plasma spiked with 1 mg/mL PSS and 0.5 mg/mL d-glucuronic acid and (**D**) plasma sample obtained at 30 min after i.g. administration of 50 mg/kg of PSS-NP from a rat. Peaks: 1, PSS; 2, d-glucuronic acid; 3, the glucose in plasma.

The specificity of the method was investigated for the assessment of potential interferences of PSS and IS from endogenous substances. Typical HPLC chromatograms of PSS after extraction from plasma are shown in Figure 3. PSS peaks were well shaped and no interfering peaks of endogenous compounds were found at the retention time of PSS or IS. These results indicated that the assay had adequate specificity.

#### 2.2.2. Linearity and Sensitivity

All calibration curves were found to be linear over the calibration range of 1–500 μg/mL. The mean (±SD) regression equation for the calibration curve in plasma was *A* = (0.0024 ± 5 × 10^−5^) × *C* + (0.0048 ± 0.0017), *r*^2^ = 0.9983 ± 0.0002, *A* represents the peak area ratio of PSS to IS and *C* represents the concentration of PSS in plasma. The LLOD and LLOQ of PSS were found to be 250 ng/mL and 1 μg/mL, which indicated the sensitivity of the method.

#### 2.2.3. Recovery

The extraction recoveries of PSS obtained from plasma at concentrations of 3, 200 and 400 μg/mL were 79.76% ± 2.13%, 83.39% ± 1.68% and 87.39% ± 6.29%, respectively. The recovery was consistent and reproducible, and it was possible to assure the accuracy and precision of the quantitative measurement of PSS in plasma.

#### 2.2.4. Precision and Accuracy

The intra-day and inter-day precisions and accuracies of the QC samples (3, 200 and 400 μg/mL) are listed in Table 1. The intra-day CV% at three concentrations of PSS were 5.94%, 2.87% and 0.67% (*n* = 5), respectively. The inter-day CV% at the above concentrations were 5.59%, 2.09% and 0.69% (*n* = 5), respectively. The data obtained were within the acceptable limit which indicated that the method was precise and accurate.

**Table 1:** Intra- and inter-day precision and accuracy of propylene glycol alginate sodium sulfate (PSS) in rat plasma (*n* = 5).

Concentration (μg/mL)	Intra-Day Precision and Accuracy			Inter-Day Precision and Accuracy		
	Mean ± SD (μg/mL)	Precision (% CV)	Accuracy (% bias)	Mean ± SD (μg/mL)	Precision (% CV)	Accuracy (% bias)
3	3.03 ± 0.18	5.94	1.00	3.04 ± 0.17	5.59	1.33
200	203.07 ± 5.83	2.87	1.54	201.04 ± 4.21	2.09	0.52
400	403.45 ± 2.72	0.67	0.87	402.55 ± 2.78	0.69	0.64

#### 2.2.5. Stability

The results of the long-term stability studies of PSS in plasma samples stored at 4 °C and −50 °C are listed in Table 2. The means of the results of tested samples were within the acceptable criteria of ±15%. This indicated that there was no significant degradation of PSS in the plasma sample and PSS was stable under the experimental conditions of the analytical runs.

**Table 2:** Stability of PSS in rat plasma at different temperatures (*n* = 5).

*T* (°C)	Concentration (μg/mL)	Mean ± SD	% CV	% Bias
4	3	2.78 ± 0.06	2.16	−7.33
	200	192.76 ± 12.21	6.33	−3.62
	400	394.77 ± 14.77	3.74	−1.31
−50	3	2.82 ± 0.22	7.80	−6.00
	200	198.83 ± 10.81	5.44	−0.59
	400	395.63 ± 12.42	3.14	−1.09

### 2.3. Application of the Developed HPLC Method to Pharmacokinetics Study

**Figure 4:**
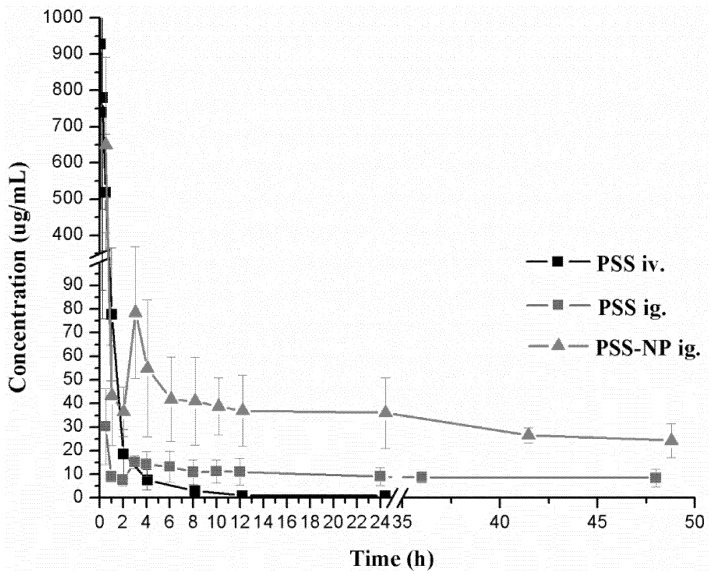
Mean plasma concentration-time curves of PSS in rats after a single intravenous injection of PSS and a single oral gavage of PSS and PSS-NP, respectively. Each data point represents the mean ± standard deviation (*n* = 7).

The validated method described above was successfully applied to the determination of PSS in rat plasma. The plasma concentration-time curves are shown in Figure 4. The relevant pharmacokinetic parameters, including *T*_1/2_, *T*_max_, *C*_max_, Cl, Vd, MRT and AUC were determined using the noncompartmental analysis (NCA) of the PKSolver software and listed in Table 3. 

**Table 3:** Pharmacokinetic parameters (mean ± SD, *n* = 7) of PSS after a single intravenous injection of PSS, a single oral gavage of PSS and PSS-NP, respectively.

Parameters	PSS		PSS-NP
	i.v.	i.g.	i.g.
*T*_1/2_ (h)	3.11 ± 0.62	35.96 ± 16.65	56.23 ± 17.46
*T*_max_ (h)	-	0.50 ± 0.00	0.50 ± 0.00
*C*_max_ (μg/mL)	1217.21 ± 361.74	22.91 ± 4.67	31.70 ± 15.30
Cl (L/(h·kg))	0.020 ± 0.0039	0.12 ± 0.032	0.054 ± 0.028 ∗
Vd (L/kg)	0.087 ± 0.021	5.59 ± 1.96	3.86 ± 1.17
MRT (h)	2.59 ± 0.71	55.96 ± 23.30	84.67 ± 22.67
AUC_0–*t*_ (h·μg/mL)	1315.52 ± 314.33	251.22 ± 30.25	477.57 ± 169.82 ∗

∗ *p* < 0.05, compared with PSS (i.g.).

After a single oral gavage of 50 mg/kg and a single intravenous injection of 25 mg/kg of PSS, the concentrations of PSS in plasma were monitored up to 48 h after oral administration and 24 h after intravenous injection. According to the pharmacokinetic parameters of PSS, the elimination half-life time (*T*_1/2_) was 35.96 ± 16.65 h after oral administration, while 3.11 ± 0.62 h after intravenous injection. The areas under the plasma concentration-time curve (AUC) of PSS were calculated to be 251.22 ± 30.25 (h·μg/mL), 1315.52 ± 314.33 (h·μg/mL) after oral and intravenous administration. The time to peak concentration (*T*_max_) was 0.50 h after a single oral administration, which indicated that the oral absorption of PSS was rapid. All the pharmacokinetic parameters in this study were similar to the ones obtained previously by the fluorescent labeling method. Taking into account the simplicity of the method and the suitability for human pharmacokinetic study, the HPLC method with postcolumn fluorescence detection was shown to be the optimal method to determine PSS and to study its pharmacokinetic characters in biological samples.

A comparative pharmacokinetic study between PSS and PSS-NP was performed by determining the concentrations of PSS in plasma up to 48 h after a single oral gavage administration (50 mg/kg). There was a significant difference (*p* < 0.05) in the pharmacokinetic parameters between the groups of PSS and PSS-NP. Compared with PSS, the maximum plasma concentration (*C*_max_) of PSS encapsulated into the nanoparticles was higher. In addition, *T*_1/2_ of PSS-NP was longer than that of PSS, consistent with the result of mean residence time (MRT), which indicated that the sustained release profile of PSS from nanoparticles led to longer circulation time in the plasma [18,19]. The plasma clearance (Cl) of PSS-NP was significantly smaller than that of PSS which showed that nanoparticles could protect PSS from rapid elimination *in vivo*. Compared with PSS, the area under the plasma concentration-time curve (AUC) of PSS-NP was significantly increased and the relative bioavailability was calculated to be 190.10%. The above results showed that PSS-loaded PLGA nanoparticles displayed improved pharmacokinetic profiles. In order to improve the bioavailability of PSS, the nanoparticle formulations of PSS will be studied and developed further in the future.

## 3. Experimental

### 3.1. Chemicals and Materials

PSS was provided by Qingdao Lantai Pharmaceutical Co., Ltd. Poly (lactic-*co*-glycolic acid) (PLGA, 50/50, average Mw 70 kDa) was purchased from Shandong Institute of Medical Devices. Polyvinyl alcohol (PVA, viscosity: 11–14 cp), Guanidine hydrochloride and d-glucuronic acid were purchased from Sigma-Aldrich (St. Louis, MO, USA). Ultra-0.5 centrifugal filters (30 kDa, MWCO) were purchased from Millipore Corporation (USA). All other chemicals were of analytical reagent grade.

### 3.2. Animals

Healthy Wistar rats (male and female), weighing 180–220 g, were obtained from Qingdao Institute for Drug Control (Qingdao, Shandong Province, China, SCXK(LU)20090007). All the rats were caged individually under controlled environmental conditions (room temperature 23 ± 2 °C, humidity 55% ± 10%, 12 h light and 12 h dark-cycle with commercial food and free water) and allowed to acclimate one week before treatment. All studies were in compliance with the Guidelines for the Care and Use of Laboratory Animals and approved by Institutional Animal Care and Use Committee in XBL-China.

### 3.3. Chromatographic and Apparatus Conditions

**Figure 5:**
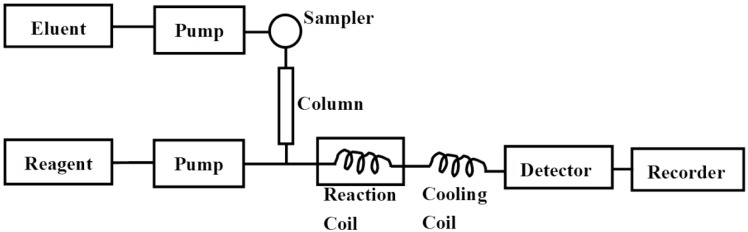
Flow diagram of the postcolumn HPLC system for determination of PSS. Eluent, 0.1 M sodium sulfate; reagent, 0.5 M sodium hydroxide (NaOH) solution containing 0.1 M guanidine hydrochloride (flow rate, 0.3 mL/min); reaction temperature, 120 °C; reaction coil, 0.5 mm i.d. × 10 m; cooling coil, 0.25 mm i.d. × 3 m; detection, excitation 250 nm and emission 435 nm; column, TSKgel G2500 PWxL column (7.8 × 300 mm, TOSOH, Japan) at 30 °C.

The postcolumn HPLC analysis was performed on a Dionex UltiMate™ 3000 HPLC system (Sunnyvale, CA, USA), which was equipped with a pump (DGP-3600SD), an autosampler (WPS-3000SL), a column compartment (TCC-300RS) and a fluorescence detector (FLD-3100) (all from Dionex UltiMate™ 3000, USA). The chromatographic separation was performed on a TSKgel G2500 PWxL column (7.8 × 300 mm, TOSOH, Japan) at 30 °C. The mobile phase was 0.1 M sodium sulfate at a flow rate of 0.5 mL/min. In the postcolumn procedure, a 0.5 M sodium hydroxide (NaOH) solution containing 0.1 M guanidine hydrochloride was passed through polytetrafluoroethylene (PTFE) reaction coil (0.5 mm i.d. × 10 m) thermostated at 120 °C and then the mixture was added to the column effluent at a flow rate of 0.3 mL/min. Subsequently, a 0.5 M sodium hydroxide solution at a flow rate of 0.3 mL/min was added to cool down the mixture in another PTFE coil (0.25 mm i.d. × 3 m) with an ice bath. Fluorescence Detection was carried out at an excitation wavelength (λex) of 250 nm and an emission wavelength (λem) of 435 nm (Figure 5). 

### 3.4. Preparation of Calibration Standards and Quality Control Samples

The stock solutions of PSS (3 mg/mL) and IS (3 mg/mL) were freshly prepared in water and kept at 4 °C before use. Working solutions of PSS were prepared by diluting with water for further concentration series of 1500, 750, 300, 150, 30, 15, 6, 3 μg/mL. Calibration work solutions were prepared by spiking 50 μL of the appropriate working solutions and 10 μL of IS solution to 100 μL of blank plasma with concentrations ranging from 1 to 500 μg/mL. The quality control (QC) samples including low (3 μg/mL), middle (200 μg/mL) and high (400 μg/mL) concentrations of standards were prepared in the same way as the calibration standards. All calibration work solutions and QC samples were stored at −50 °C until analysis.

### 3.5. Sample Preparation

All plasma samples, spiked calibration work solutions, or spiked QC samples were treated in the same manner as described below. To a 100 μL portion of plasma sample, 50 μL of water and 10 μL of IS solution were added. Then the mixtures were vortexed for 3 min and transferred into a 30 kDa Ultra 0.5 centrifugal filter device. After centrifugation at 10,000× *g* for 20 min, the aliquot (20 μL) of the supernatant was submitted to the HPLC system.

### 3.6. Method Validation

#### 3.6.1. Specificity

The specificity of the method was investigated by comparing chromatograms of blank rat plasma obtained from six rats, the mixture of PSS and IS, the blank plasma spiked with PSS and IS, and plasma samples obtained from PSS-NP in its pharmacokinetic studies. There should be no interference from endogenous or exogenous materials observed at the retention time of PSS and the IS.

#### 3.6.2. Linearity and Sensitivity

An eight-point calibration curve with final concentrations of 1, 2, 5, 10, 50, 100, 250 and 500 μg/mL was constructed by plotting the peak area ratios of PSS to IS against the concentrations of PSS. The linearity of the calibration curve was evaluated by linear regression analysis. One calibration curve was constructed for each analysis day using freshly prepared calibration standards. The lower limit of quantitation (LLOQ) was determined at the lowest concentration on the calibration curve with a relative standard deviation (RSD) of less than 20%. The LLOQ was evaluated by analyzing samples in six replicates on three consecutive days [20]. The limit of detection (LOD) was defined as the lowest concentration level resulting from a signal-to-noise ratio of 3:1.

#### 3.6.3. Recovery

The absolute recovery of PSS from rat plasma was calculated at concentrations of 3, 200 and 400 μg/mL by comparing the peak area ratios of extracted samples with ones in which the compound was spiked directly to the mobile phase. Recoveries at three QC concentration levels for plasma were examined at least five times.

#### 3.6.4. Precision and Accuracy

The precision and accuracy of the method were determined by analyzing QC samples at three different concentrations (3, 200 and 400 μg/mL). Intra-day precision and accuracy were assessed from replicate analysis (*n* = 5) of QC samples at each concentration level on the same day. Inter-day precision and accuracy were evaluated from the analysis of the same QC samples on five consecutive days (*n* = 5). Precision was determined by calculating the CV for each replicates and accuracy was assessed by calculating the percentage bias from the nominal concentration. The intra-day and inter-day precisions were required to be within ±15% relative standard deviation (RSD) and the accuracy to be within ±15% bias from the nominal values.

#### 3.6.5. Stability

The stability of PSS in plasma was evaluated by analyzing three QC samples at different concentration levels (3, 200 and 400 μg/mL), which were stored at 4 °C or −50 °C for five days. All the samples were analyzed as described above. The concentrations obtained were compared with the theoretical value of the QC samples to determine the long-term stability of PSS in rat plasma. The samples were considered to be stable if the percentage change (bias) in the concentration of the stability samples was not more than ±15% for higher concentrations and below ±20% for lower concentrations.

### 3.7. Application to Pharmacokinetic Study of PSS-Loaded Nanoparticles

An *in vivo* pharmacokinetic study was undertaken to investigate the pharmacokinetic behaviors after the administration of the PSS-NP suspension and PSS solution.

#### 3.7.1. Animal Administration and Sampling

Twenty-one rats were randomly divided into three groups, oral administration group of PSS-NP, oral administration group of PSS and intravenous injection group of PSS. All the rats were fasted overnight prior to dosing. PSS was dissolved in sterile saline and administered to the rats by a single oral gavage (50 mg/kg) and a single injection via the tail vein (25 mg/kg), while PSS-NP was prepared by suspending freeze-dried powders in sterile saline by a single oral gavage at the same dose of PSS. The blood samples (about 0.3 mL) collected from the tip of the tail at predetermined time intervals up to 48 h after oral administration and 24 h after intravenous injection were placed into sodium citrate micro-centrifuge tubes. The blood samples were centrifuged at 3000 rpm for 15 min and the separated plasma (100 μL) was stored at −50 °C until analysis. The following procedures were similar to those which were described in Section 3.5. All the plasma samples were assayed within two days of the administration.

#### 3.7.2. Calculations and Statistics

HPLC results of samples were analyzed with Chromeleon software (Sunnyvale, CA, USA). PKSolver software [21,22] was employed to analyze the plasma concentration-time data with noncompartmental analysis (NCA). The area under the concentration–time curve (AUC_0–t_) from zero to the last time point, mean residence time (MRT), elimination half-life time (*T*_1/2_), volume of distribution (Vd), plasma clearance (Cl), peak concentration (*C*_max_) and the time to peak concentration (*T*_max_) of the drug were all obtained.

Data were presented as mean ± standard deviation (SD). One-way analysis of variance (ANOVA) test was performed on the data to assess the impact of the formulation variables on the results (*n* ≥ 3). Differences were considered statistically signiﬁcant at *p* < 0.05.

## 4. Conclusions

In this study, a highly sensitive and effective HPLC method with postcolumn fluorescence detection was developed to determine PSS in rat plasma. This method, using guanidine hydrochloride as the fluorescence derivatization reagent, was validated to confirm high accuracy, precision, and reproducibility. The pharmacokinetic parameters of PSS were characterized and the LLOD was 250 ng/mL which indicated that this method was of high sensitivity. The method was successfully applied to the pharmacokinetic analysis of PSS and PSS-loaded PLGA nanoparticle suspensions in rats. The results showed that, compared with PSS, PSS-NP could prolong the residence time of PSS in the plasma, enlarge the AUC, lower plasma clearance, and significantly improve the relative bioavailability. PSS-loaded PLGA nanoparticles can improve pharmacokinetic profiles *in vivo* and will be studied and developed further in the future.
